# Detection of North American orthopoxviruses by real time-PCR

**DOI:** 10.1186/1743-422X-8-313

**Published:** 2011-06-20

**Authors:** Nadia F Gallardo-Romero, Andres Velasco-Villa, Sonja L Weiss, Ginny L Emerson, Darin S Carroll, Christine M Hughes, Yu Li, Kevin L Karem, Inger K Damon, Victoria A Olson

**Affiliations:** 1 Centers for Disease Control and Prevention, National Center for Emerging and Zoonotic Infectious Diseases, Division of High-Consequence Pathogens and Pathology, Poxvirus and Rabies Branch, Atlanta, GA 30333, USA

**Keywords:** orthopoxviruses, North American orthopoxviruses, myristylated protein, real time PCR

## Abstract

The prevalence of North American orthopoxviruses in nature is unknown and may be more difficult to ascertain due to wide spread use of vaccinia virus recombinant vaccines in the wild. A real time PCR assay was developed to allow for highly sensitive and specific detection of North American orthopoxvirus DNA in animal tissues and bodily fluids. This method is based on the amplification of a 156 bp sequence within a myristylated protein, highly conserved within the North American orthopoxviruses but distinct from orthologous genes present in other orthopoxviruses. The analytical sensitivity was 1.1 fg for *Volepox virus *DNA, 1.99 fg for *Skunkpox virus *DNA, and 6.4 fg for *Raccoonpox virus *DNA with a 95% confidence interval. Our assay did not cross-react with other orthopoxviruses or ten diverse representatives of the *Chordopoxvirinae *subfamily. This new assay showed more sensitivity than tissue culture tests, and was capable of differentiating North American orthopoxviruses from other members of *Orthopoxvirus*. Thus, our assay is a promising tool for highly sensitive and specific detection of North American orthopoxviruses in the United States and abroad.

## Background

The family *Poxviridae *is divided in two subfamilies: *Entomopoxvirinae*, which infect insects, and *Chordopoxvirinae*, which infect vertebrates[1]. Genera of the subfamily *Chordopoxvirinae *that may cause human infections include *Orthopoxvirus*, *Parapoxvirus, Molluscipoxvirus *and *Yatapoxvirus*[1,2]. The genus *Orthopoxvirus *(OPXV) is the most relevant in terms of human public health concerns and includes viruses that have been associated with severe febrile, rash illness in humans. Its members include: *Variola virus*, a solely human pathogen and the etiological agent of smallpox[2-4]; *Monkeypox virus*, a zoonotic disease that causes a generalized rash similar to smallpox with up to 10% case fatality rate[5]; *Vaccinia virus *(VACV), the smallpox vaccine which can produce generalized illness in immunocompromised individuals[6]; and *Cowpox virus*, another zoonotic disease which can cause generalized illness in immunocompromised individuals[6,7]. In immunocompetent individuals, infection with vaccinia or cowpox viruses usually only results in localized rash illness. The other viruses within this genus are not currently known to cause human disease.

The last few decades have seen the description of three orthopoxviruses (OPXVs) from North America named after the animal species in which they were originally isolated: *Raccoonpox virus, Volepox virus*, and *Skunkpox virus*[8-11]. These OPXVs are collectively referred to as North American OPXVs (NA OPXVs). Subsequent work has determined that the NA OPXVs species are a monophyletic group that is the most genetically divergent within the OPXV genus[11]. *Raccoonpox virus *(RCNV) was isolated for the first time in Maryland, 1961, from two out of 97 healthy looking raccoons (*Procyon lotor*). In the same study, an orthopoxvirus seroprevalence rate of 23% (22/92) was observed[8]. *Volepox virus *(VPXV) was first isolated from a California vole (*Microtus californicus*) foot scab in 1985[12], and later from a Piñon mouse (*Peromyscus truei*) scab in 1988[10], both in San Mateo county, California. *Skunkpox virus *(SKPV) has been isolated only once from an ill skunk in Washington in 1978[11]. Few studies have been conducted regarding the ecology, pathology and pathogenicity of these NA OPXVs[9-14], but their prevalence and role as etiological agents of potential zoonotic diseases remain unknown.

The development of a highly specific and sensitive assay for detection of NA OPXVs is critical to the understanding of NA OPXVs incidence and prevalence in North American mammals. In addition, the effect of NA OPXVs endemicity on the immunization of wildlife populations against other infectious diseases, using VACV vectored vaccines, is unknown. Orthopoxvirus-vectored recombinant wildlife vaccines [e.g. rabies, plague[14-17]], could have the potential to recombine with NA OPXVs in nature. The use of this assay will enable detailed studies of NA OPXVs prevalence, pathology and pathogenesis in their putative host species.

Additional studies using these specific determinations of NA OPXVs prevalence and incidence rate will inform subsequent studies evaluating the possible interference of NA OPXVs disease and immunity, with disease control strategies in which orthopoxvirus-vectored vaccines target the same susceptible wildlife species.

## Methods

### Alignment and phylogenetic reconstruction

**Figure 1:**
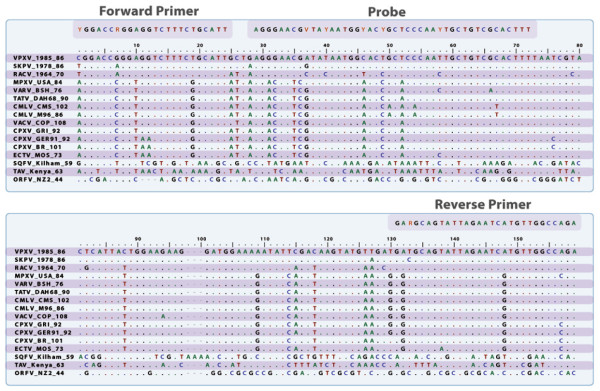
**Alignment of the 156 bp target area used to design the primers and probe of G9R NA OPXV rt-PCR assay**.

Amino acid sequences of the G9R gene were aligned using the ClustalW accessory in BioEdit (Figure 1) and translated back into the original nucleotide sequence for phylogenetic analysis. A heuristic tree search was carried out in PAUP* 4.0b10 over 1000 replicates.

**Figure 2:**
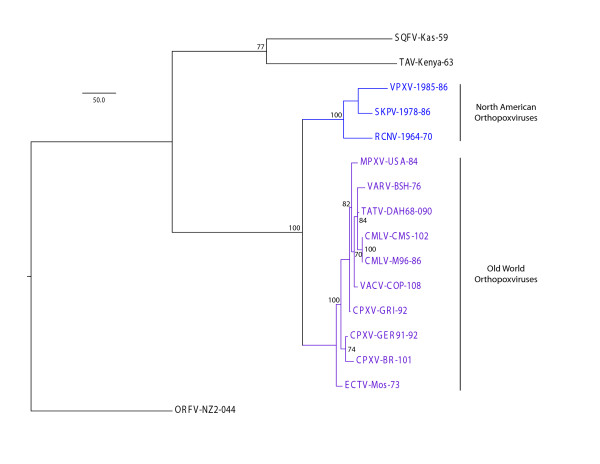
**One of the two most parsimonious trees**. Bootstrap values over 50% are shown at each relevant node. Branch lengths are drawn to scale.

Bootstrap values were calculated from 1000 bootstrap replicates of 1000 random addition sequence replicates each. One of two most parsimonious trees is illustrated in Figure 2.

### Primer, probe design and assay conditions

We designed a real time PCR (rt-PCR) assay based on TaqMan^® ^chemistry and technology using the VPXV 086 gene, SKPV 086 gene and RCNV 070 gene (ortholog of the VACV Copenhagen *G9R*). The targeted gene encodes for the myristylated protein, which is an essential component for cell membrane fusion and entry[18]. The orthologous genes for all viruses included in this study were aligned using BioEdit to pick the target area on the basis of conservation and GC content. The primers G9R-NA OPXV forward (5'YGG-ACC-RGG-AGG-TCT-TTC-TGC-ATT 3'), G9R-NA OPXV reverse (5'TCT-GGC-CAA-CAT-GAT-TCT-AAT-ACT-GCR-TC 3'), and G9R-NA OPXV probe (5'FAM AG-GGA-ACG-VTA-YAA-TGG-YAC-YGC-TCC-CAA-YTG-CTG-TCG-CAC-TTT-BHQ1 3') were designated to amplify a 156 bp fragment from a highly conserved region of the gene (Figure 1). Primers and probe were synthesized in the Biotechnology Core facility (CDC, Atlanta, GA), utilizing standard phosphoramidite chemistry. The nomenclature for the degenerated positions in the primers and probe designed was based on IUPAC. The probe contained the fluorophore (FAM) at the 5' end and the black hole quencher at the 3' end. DNA amplification was carried out using default rt-PCR thermal conditions for the ABI7900 (Applied Biosystem, Foster City, Ca), briefly: one cycle of 95°C for 10 minutes, followed by 40 cycles of 95°C for 15 seconds and 60°C for 1 minute. PCR amplification is based on fluorescent emission after annealing/elongation (60°C). The assay was performed in a final volume of 25 ul per well containing 12.5 ul of TaqMan^® ^Universal PCR Master Mix (Applied Biosystem, New Jersey, USA), 4.5 ul of deionized distilled water, 1 ul of each forward and reverse primer at a concentration of 20 uM, 1 ul of probe at a concentration of 10 uM, and 5 ul of template DNA.

### Analytical sensitivity and specificity of G9R-NA OPXV rt-PCR assay

A total of thirteen chordopoxviruses, which comprised seven old world OPXVs, three NA OPXVs, and three from different genera, were included to test the analytical specificity of the assay (Table 1). The latter three included one *Parapoxvirus*, one *Leporipoxvirus *and one *Yatapoxvirus*. All viruses in the panel (with the exception of the squirrel fibroma virus, which was a crude cell culture extract), were propagated via cell culture and purified by two sucrose cushions[19-21]. Total viral DNA was extracted using Qiagen tissue kits on the BioRobot^® ^EZ1 workstation, according to the manufacturer's instructions, and later quantified by spectrophotometry using Qubit™ Quantitation Platform by Invitrogen™. All viruses in the panel were serially diluted with deionized distilled water until obtaining appropriate working concentrations. To determine analytical specificity of our assay, the total DNA concentration was adjusted to 2 ng per 5 ul for all the thirteen viruses in the panel, and the rt-PCR assay was run in triplicate for each sample.

**Table 1:** Panel of viral DNA used to validate the G9R-NA OPXV rt-PCR assay

Old World viruses	Sample ID	**Accession No**.	Ortholog	**Viral Prep**.	2 ng*
*Camelpox*	CMLV-V78 I	Not available	Not available	Pure	ND
*Cowpox*	CPXV-Sweden	Not available	Not available	Pure	ND
*Ectromelia*	ECTV-MOS	NC_004105	73	Pure	ND
*Monkeypox*	MPXV-USA	DQ011157	84	Pure	ND
*Orf*	ORFV-NZ2	DQ184476	44	Pure	ND
*Squirrel Fibroma*	SQFV-Kilham	Not available	59	Crude	ND
*Tanapox*	TAV-Kenya	NC_009888	63	Pure	ND
*Taterapox*	TATV-DAH68	NC_008291	90	Pure	ND
*Vaccinia*	VACV-COP	M35027	108	Pure	ND
*Variola*	VARV-BSH	L22579	76	Pure	ND
**North American viruses**					

*Raccoonpox*	RCNV-1964	FJ807746-54	70	Pure	17
*Skunkpox*	SKPV-1978	FJ807755-63	86	Pure	17
*Volepox*	VPXV-1985	FJ807737-45	86	Pure	16

*The average of the cycle when fluorescence crossed the threshold (Ct) for each positive sample is shown. ND, not detected.

Analytical sensitivity was determined by using DNA of the three NA OPXVs at seven different concentrations, 40 fg, 4 fg, 2 fg, 1 fg, 0.5 fg, 0.25 fg, and .125 fg/ul. The rt-PCR for each concentration was run in 24 replicates. A statistical probit analysis, a non-linear regression model, using commercial software SAS 9.2 (SAS Institute, Cary, NC, USA) was done to determine the detection limit of each assay with 95% confidence.

### Animal specimen collection and preparation

*Peromyscus californicus *(Peromyscus Genetic Stock Center, University of South Carolina) were infected intranasally with 10 ul of volepox virus at a concentration of 1.6 × 10^3 ^PFU or mock infected with 10 ul of phosphate-buffered saline (PBS). Pre-screening of the animals included evaluation of anti-VPXV and anti-VACV immunoglobulin type G by modified ELISA with 1:100 dilution of animal serum, and orthopoxviral DNA in EDTA blood, oral swab, ocular swab and anal swabs by rt-PCR as previously described[22,23]. Specimens taken from live animals (blood, oral swab, ocular swab, and anal swab), and during necropsy (spleen, gonad, kidney, submandibular lymph node, supradrenal gland, brain, liver and lung) were performed/collected in accordance with CDC Institutional Animal Care and Use Committee (IACUC) guidelines under the approved protocol 2126 CARMOUC. In some animals, additional organs were taken at necropsy if lesions or signs of disease were observed (skin, tongue, bladder, intestine and/or pancreas) (Table 2). A total of 187 samples, from different tissues and bodily fluids, were collected from animals with clear signs of disease (for example: conjunctivitis, ruffled hair, skin lesions, crusty noses, apathy and/or swollen faces) on days 6, 7 and 8 post-infection as well as 45 tissue samples and bodily fluids from healthy, never infected *Peromyscus californicus*. Swabs were hydrated for 5 minutes with 400 ul of PBS, and transferred to SETS tubes (Swab Extraction Tube System, Roche, Catalog No. 3315568). The SETS tubes were centrifuged in the VWR Galaxy Mini centrifuge (6000 rpm) for 1 minute to collect the eluant for DNA extraction and cell culture. The tissue samples were suspended in 1 ml of PBS and a sterile SPEX bead was added. The GenoGrinder 2000 (SPEX Sample Prep) was used following the manufacturer's instructions to triturate the tissue for later cell culture and DNA extraction using the Qiagen tissue kits on the BioRobot^® ^EZ1 workstation, according to the manufacturer's instructions.

**Table 2:** Samples from *P. californicus *either negative control (un) or infected with volepox virus (inf) were tested with G9R-NA OPXV rt-PCR assay

Specimen	Day 6		Day 7		Day 8		Total No. of specimens	
	(Inf)	(un)	(inf)	(un)	(inf)	(un)	(inf)	(un)
Blood	4	1	3	1	2	1	9	3
Oral swab	6	1	5	1	7	1	18	3
Ocular swab	6	1	8	1	2	1	13	3
Anal swab	5~	1	3~	1	2	1	13	3
Spleen	6	1	5	1	2	1	13	3
Gonad	6	1	5	1	2	1	13	3
Kidney	6	1	5	1	2	1	13	3
Lymph node	6	1	5	1	2	1	13	3
Supradrenal	6	1	4	1	2	1	12	3
Brain	6	1	5	1	2	1	14	3
Liver	6	1	5	1	2	1	14	3
Lung	6	1	5	1	2	1	14	3
Skin lesion	5	1	2	1	10	1	14	3
Tognue lesion	5	1	2	1	NA	1	7	3
Intestine lesion	NA	1	2	1	NA	1	2	3
Bladder	NA	NA	2	NA	1	NA	3	NA
Pancreas	NA	NA	1	NA	1	NA	2	NA

Number of positive/tested	79/80	0/15	64/66	0/15	41/41	0/15	184/187	0/45

~DNA was detected in all samples from infected animals (inf) but 3 anal swabs (1 from Day 6 and 2 from Day 7).

DNA was not detected in any negative control (un). NA, not applicable.

### Detection of viable OPXV in animal samples through tissue cell culture techniques

BSC-40 cell monolayers (African green monkey kidney cell line) were inoculated with 10-fold dilutions of sonicated tissue extract or swab elute. Infected cells were incubated at 36°C in a 6% CO_2 _atmosphere in semi solid medium (RPMI + 1% Carboxymethylcellulose). Cell infection was monitored microscopically by observation of OPXV specific cytopathic effect (CPE). At 48 h post inoculation, cells were stained with crystal violet to reveal plaques to determine the viral titer (PFU/ml). A total of 187 putative positive, and 45 negative samples were examined for the presence of viable virus by cell culture isolation. These samples were also used for determining analytical sensitivity and specificity of the rt-PCR.

## Results

### Analytical specificity of G9R-NA OPXV rt-PCR assay

The orthopoxviruses are all very closely related[11]. Yet the NA OPXVs cluster into their own phylogenetic subclade, as shown in the alignment of the myristylated protein ortholog (Figure 2). Our NA OPXV rt-PCR assay was validated using at least one representative strain of each species of OPXV as well as one strain from the genera *Parapoxvirus*, *Leporipoxvirus*, and *Yatapoxvirus *(Table 1). The assay consistently detected all three NA OPXVs at 2 ng total DNA (equivalent to > 8-9 million virus genomes), with threshold cycle (Ct) values ranging from 16 to 17(Table 1). The purity of these viral preparations was verified using a real-time PCR assay to detect contaminating cellular DNA (RNase P), which did not cross-react with any of the NA OPXVs DNA preparations (data not shown). The NA OPXV rt-PCR assay demonstrated specificity for NA OPXVs, not reacting with the other seven OPXVs or three non-orthopoxvirus members of *Chordopoxviridae *when tested at similar concentrations (Table 1).

### Analytical sensitivity of G9R-NA OPXV rt-PCR assay

**Figure 3:**
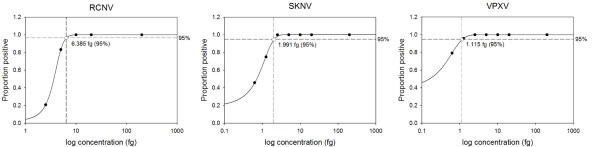
**Amount of viral DNA detected by the G9R NA OPXV rt-PCR assay with 95% confidence based on the probit statistical analysis**.

To determine the analytical sensitivity of the assay for each of the NA OPXVs, 24 replicates of seven different concentrations were conducted with the NA OPXV rt-PCR assay. The assay was least sensitive for RCNV; DNA was detected in all 24 replicates down to 10 fg of DNA (Table 3). For 5 fg of total DNA, 20 out of 24 replicates yielded positive results, and for 2.5 fg, five out of 24 replicates were detected as positive. The assay showed higher sensitivity for SKPV DNA, with positive results in all 24 replicates down to 2.5 fg of total DNA (Table 3). At 1.25 fg, 18 of 24 replicates were detected as positive, and at 0.625 fg, eleven of 24 replicates were positive. The assay proved its highest sensitivity for VPXV DNA. All 24 replicates at concentrations as low as 2.5 fg were detected (Table 3). At 1.25 fg of total VPXV DNA, the assay detects 23 of the 24 replicates, and at 0.625 fg, 19 of the replicates were positive. The average Ct values from all positive samples are shown in Table 3. Based on RNaseP assay results, we can confidently calculate the number of viral genomes detected based on mass, since there was no detectable cellular DNA contamination in our preparations. The statistical analysis (probit) indicated that the assay is capable of detecting with 95% confidence, 1.1 fg of VPXV DNA (~5 genomes), 1.99 fg of SKPV DNA (~8 genomes) and 6.4 fg of RCNV total DNA (~29 genomes) under the cycling conditions, primers and probes described (Figure 3).

**Table 3:** G9R-NA OPXV assay sensitivity to North American OPXV DNA

Virus	DNA (fg)	200	20	10	5	2.5	1.25	0.625
*Raccoonpox*	Pure	33	36.5	37.9	38.9 *(20)*	39.4 *(5)*	NT	NT
*Skunkpox*	Pure	28.9	32.7	34	35	36	37.8 *(18)*	39.2 *(11)*
*Volepox*	Pure	30.4	33.9	35.2	36.1	37.4	38.8 *(23)*	39.4 *(19)*

The average of the cycle when fluorescence crossed the threshold (Ct) for each positive sample is shown.

NT, not tested *(number of sample detected from 24 replicates)*. DNA amounts are shown in fg.

### Specificity and sensitivity of detection of NA OPXV DNA in animal samples by rt-PCR

Analysis of all samples (blood, oral swab, ocular swab and anal swabs) taken prior to infection tested negative for OPXV DNA by rt-PCR, and anti-VPXV and anti-VACV IgG by modified ELISA[22,23](data not included in the assay evaluation). One hundred and eighty four out of 187 specimens collected at days 6, 7 and 8 post-infection from different tissues and bodily fluids generated Ct values in the positive range (between 15 and 40) for our NA OPXV rt-PCR assay, indicating the presence of DNA specific for a NA OPXV (Table 2). Only three anal swabs out of 13 taken were negative, and may contain levels of viral DNA under the detection threshold for our assay (1.1 fg). All 45 samples from the naïve (PBS mock infected) animals were negative by our G9R-NA OPXV rt-PCR assay (Table 2).

### Comparative detection of NA OPXV in animal samples with cell culture versus rt-PCR

One hundred and seventy-eight samples out of a total of 184 rt-PCR positive samples demonstrated detectable CPE in a single passage on BSC-40 cells. All these samples had a Ct values of 37 or below which, based on the quantification from the standard curve, would equate to a minimum of 45 genomes of VPXV DNA. Six samples out of 184 rt-PCR positives with Ct values of 38 and above were discordant with cell culture viability results; in these samples no viable virus was detected and the estimated number of genomes is less than 45. All 45 samples from naïve animals were confirmed negative by both rt-PCR and cell culture. When comparing cell culture with rt-PCR results, samples with Ct values equal or lower than 37 were positive for VPXV growth, and samples with Ct values 38 and above did not show evidence of viable VPXV.

## Discussion

North American OPXVs have been detected previously using the Low GC Pan-Pox standard end point PCR assay[24]. However, the sensitivity of this standard PCR assay is lower (~10,000 genome copies) and more time intensive than our rt-PCR assay for the detection of NA OPXVs (<30 genome copies). The G9R-NA OPXV assay is a high throughput assay better suited for screening proposes either in the field or laboratory experiments that aim to detect low levels of NA OPXV DNA. The assay presented here showed an analytical sensitivity of 1.1 fg of VPXV DNA, 1.99 fg of SKPV DNA and 6.4 fg of RCNV DNA, which is equivalent to approximately five, eight, and 29 viral genomes respectively. Our assay also demonstrated high specificity, capable of discriminating all NA OPXVs from several other members of the genus *Orthopoxvirus *and the subfamily *Chordopoxvirinae*. Since our assay does not cross-react with VACV, it is possible to determine if wild animals are potentially co-infected with a NA OPXV and a VACV vector vaccine (ex. Rabies, plague). The presence of more than one OPXV in the same individual may mask the detection of the other virus when detection assays are broadly reactive. The G9R-NA OPXV rt-PCR assay will be useful for future studies on the efficacy of VACV vector vaccines within wild animal populations where NA OPXVs are endemic.

This rt-PCR assay may also offer great utility in confident detection of NA OPXVs DNA within tissues and bodily fluid samples obtained from infected animals. Its sensitivity and specificity significantly surpasses that of tissue cell culture. Unlike tissue cell culture, our NA OPXV rt-PCR assay is capable of quickly differentiating between Eurasian OPXV and NA OPXV. Although tissue culture is still the only means to detect viable viral particles, our assay demonstrates greater sensitivity than tissue culture, most likely due to the ability to detect viral DNA from damaged viral particles or DNA remnants. Previous OPXV studies done during the US monkeypox outbreak in 2003, also found rt-PCR assays to be more sensitive than tissue culture analysis [22,25,26]. Our assay detected six samples as positive that did not contain viable virus, possibly due to compromised integrity of the viral particle by either the animal immune system or by an external factor during the sample collection or processing. A benefit of such a sensitive assay is the ability to prove that animals were infected even when viable virus is no longer present or viral isolation is unable to be performed.

## Conclusions

The sensitivity and specificity of this assay makes it a highly useful tool for detecting NA OPXV in field studies as well as a monitoring tool in pathogenesis studies. Our rt-PCR assay is a more sensitive, rapid and less labor intensive method for the specific identification of NA OPXV DNA within a sample, allowing prompt diagnosis of infection.

## Competing interests

The authors declare that they have no competing interests.

## Authors' contributions

NG-R conceived of the study, participated in its design, assay validation, sample processing, data analysis, and drafted the manuscript. AV-V performed the structural gene alignments, primer design and drafted the manuscript. SW assisted in acquiring animal samples and processing the samples. GE carried out the phylogenetic reconstructions and drafted the manuscript. DC contributed to study design and obtained funds for the research. CH performed statistical analysis. YL established the rt-PCR settings to optimize the assay. KK and ID contributed to study design and provided useful manuscript review. VO designed molecular validation of the rt-PCR assay and contributed to final manuscript version. All authors read and approved the final manuscript.
